# Ultrastructure and molecular phylogenetic position of a novel euglenozoan with extrusive episymbiotic bacteria: *Bihospites bacati *n. gen. et sp. (Symbiontida)

**DOI:** 10.1186/1471-2180-10-145

**Published:** 2010-05-19

**Authors:** Susana A Breglia, Naoji Yubuki, Mona Hoppenrath, Brian S Leander

**Affiliations:** 1 Canadian Institute for Advanced Research, Program in Integrated Microbial Biodiversity, Departments of Botany and Zoology, University of British Columbia, 6270 University Boulevard, Vancouver, BC V6T 1Z4, Canada; 2 Forschungsinstitut Senckenberg, Deutsches Zentrum für Marine Biodiversitätsforschung (DZMB), Südstrand 44, D-26382 Wilhelmshaven, Germany

## Abstract

**Background:**

Poorly understood but highly diverse microbial communities exist within anoxic and oxygen-depleted marine sediments. These communities often harbour single-celled eukaryotes that form symbiotic associations with different prokaryotes. During low tides in South-western British Columbia, Canada, vast areas of marine sand become exposed, forming tidal pools. Oxygen-depleted sediments within these pools are distinctively black at only 2-3 cm depth; these layers contain a rich variety of microorganisms, many of which are undescribed. We discovered and characterized a novel (uncultivated) lineage of heterotrophic euglenozoan within these environments using light microscopy, scanning and transmission electron microscopy, serial sectioning and ultrastructural reconstruction, and molecular phylogenetic analyses of small subunit rDNA sequences.

**Results:**

*Bihospites bacati *n. gen. et sp. is a biflagellated microbial eukaryote that lives within low-oxygen intertidal sands and dies within a few hours of exposure to atmospheric oxygen. The cells are enveloped by two different prokaryotic episymbionts: (1) rod-shaped bacteria and (2) longitudinal strings of spherical bacteria, capable of ejecting an internal, tightly wound thread. Ultrastructural data showed that *B. bacati *possesses all of the euglenozoan synapomorphies. Moreover, phylogenetic analyses of SSU rDNA sequences demonstrated that *B. bacati *groups strongly with the Symbiontida: a newly established subclade within the Euglenozoa that includes *Calkinsia aureus *and other unidentified organisms living in low-oxygen sediments. *B. bacati *also possessed novel features, such as a compact C-shaped rod apparatus encircling the nucleus, a cytostomal funnel and a distinctive cell surface organization reminiscent of the pellicle strips in phagotrophic euglenids.

**Conclusions:**

We characterized the ultrastructure and molecular phylogenetic position of *B. bacati *n. gen. et sp. Molecular phylogenetic analyses demonstrated that this species belongs to the Euglenozoa and currently branches as the earliest diverging member of the Symbiontida. This is concordant with ultrastructural features of *B. bacati *that are intermediate between *C. aureus *and phagotrophic euglenids, indicating that the most recent ancestor of the Symbiontida descended from phagotrophic euglenids. Additionally, the extrusive episymbionts in *B. bacati *are strikingly similar to so-called "epixenosomes", prokaryotes previously described in a ciliate species and identified as members of the Verrucomicrobia. These parallel symbioses increase the comparative context for understanding the origin(s) of extrusive organelles in eukaryotes and underscores how little we know about the symbiotic communities of marine benthic environments.

## Background

The Euglenozoa is a diverse group of single-celled eukaryotes consisting of three main subgroups: euglenids, kinetoplastids and diplonemids. Euglenids are united by the presence of a distinctive pellicle, a superficial system formed by four major components: the plasma membrane, a pattern of repeating proteinaceous strips that run along the length of the cell, subtending microtubules and tubular cisternae of endoplasmic reticulum [1]. The group is widely known for its photosynthetic members (e.g. *Euglena *and *Phacus*), but the majority of the species are heterotrophic (osmotrophs or phagotrophs). Photosynthetic euglenids evolved from phagotrophic ancestors with a complex feeding apparatus and a large number of pellicle strips that facilitate a characteristic peristaltic cell movement called "euglenoid movement". This combination of characters allows phagotrophic euglenids to engulf large prey cells, such as eukaryotic algae, which eventually led to the acquisition of chloroplasts via secondary endosymbiosis [2,3]. Euglenids are closely related to kinetoplastids and diplonemids. Kinetoplastids (a group that includes free-living bodonids and parasitic species such as *Trypanosoma and Leishmania*) are united by the presence of a mitochondrial inclusion of distinctively arranged DNA molecules, called a kinetoplast or kDNA [4]. Kinetoplastids and euglenids share several morphological features, such as flagella with hairs and heteromorphic paraxial rods (e.g. a proteinaceous scaffolding adjacent to the usual 9+2 axoneme) and mitochondria with paddle-shaped (discoidal) cristae [5-7]. Diplonemids, on the other hand, possess a large mitochondrion with flattened cristae and apparently lack flagellar hairs [8]. The monophyly of the Euglenozoa has been established on the basis of both molecular phylogenetic analyses and the following morphological synapomorphies: a tripartite flagellar root system, presence of heteromorphic paraxial rods and tubular extrusomes.

Environmental sequencing of oxygen depleted sediments around the world has shown that these habitats harbour a vast and unknown diversity of microbial lineages [9-14]. Phylogenetic analyses of these data have helped demonstrate the existence of several novel lineages associated with many different eukaryotic supergroups. Although these types of analyses are very effective in revealing the actual diversity of microbes living in a particular environment, these approaches also generate vast amounts of "orphan" data that cannot be linked directly to organisms known from comparative morphology. Nonetheless, some of the environmental sequences recovered from oxygen depleted environments cluster with euglenozoans in phylogenetic analyses but with no clear position within the group [9-11].

Other studies have explored and characterized the microbial diversity in oxygen-depleted environments using microscopical approaches [15-20]. This research has shown that a reoccurring feature of euglenozoans living in low oxygen environments is the presence of episymbiotic bacteria on the cell surface. Here, we report on a highly unusual (uncultivated) euglenozoan isolated from oxygen depleted marine sediments that is covered with two very different morphotypes of episymbionts. We characterized this lineage with light microscopy, SEM, comprehensive TEM, and molecular phylogenetic analyses of SSU rDNA sequences. Our data demonstrate that this organism is the earliest diverging member of the Symbiontida, which is an emerging subclade of euglenozoans composed of anaerobic and microaerophilic flagellates with a superficial layer of mitochondrion-derived organelles that associates closely with a uniform layer of episymbiotic bacteria [19]. Moreover, the comparative ultrastructural data from this novel lineage sheds considerable light onto the phylogenetic position of the Symbiontida, as a whole, within the Euglenozoa.

## Results

### General Morphology

**Figure 1:**
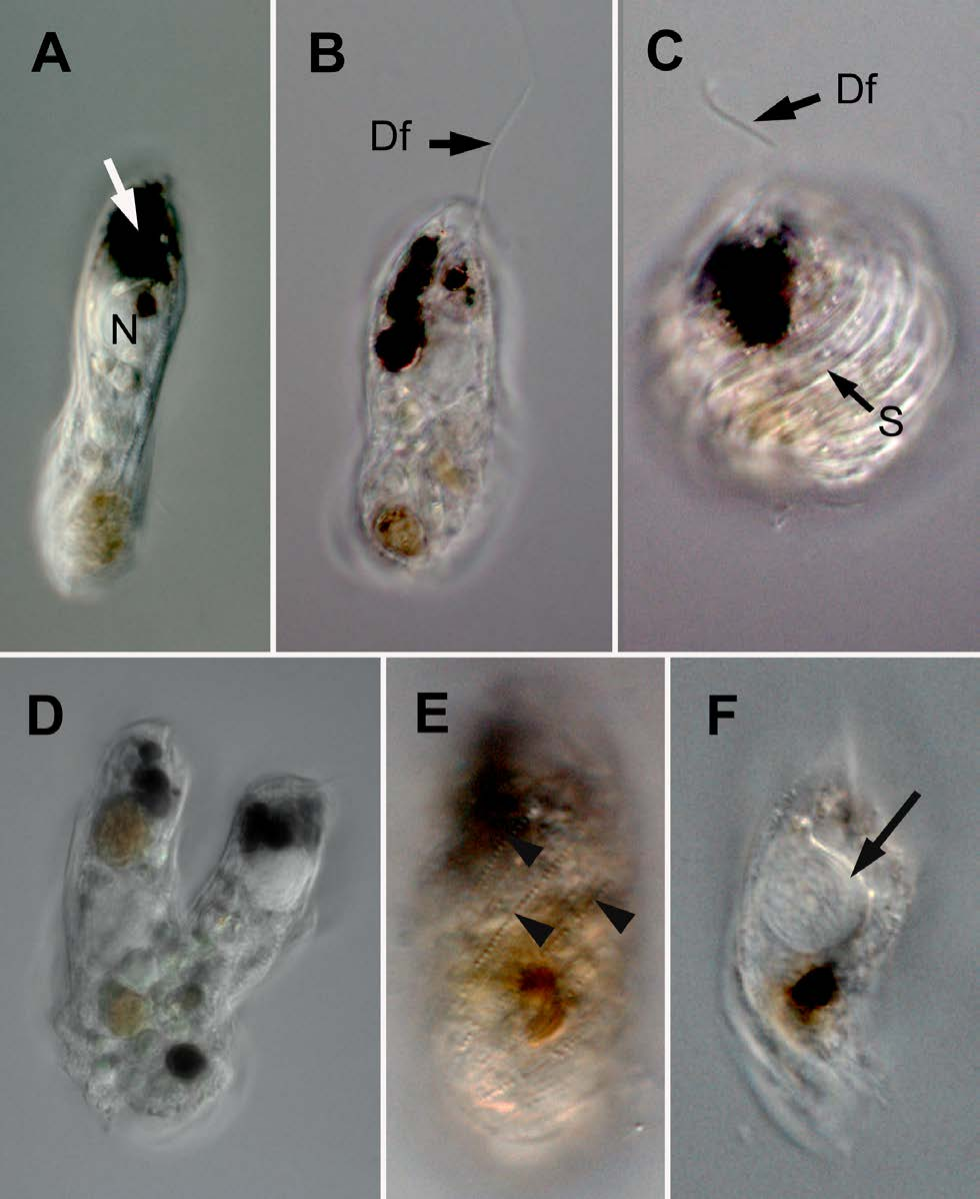
**Light micrographs (LM) of living cells of *Bihospites bacati *n. gen. et sp**. **A**. LM showing distinctive black bodies (white arrow) and the prominent nucleus (N) positioned near the anterior end of the cell. **B**. LM showing the extended dorsal flagellum (Df) that is inserted subapically. **C**. LM showing the dorsal flagellum (Df) and a contracted cell with raised helically arranged striations (S) on the surface. **D**. LM showing a cell dividing along the anteroposterior axis. **E**. LM showing rows of spherical-shaped bacterial episymbionts on the cell surface (arrowheads). **F**. LM showing the nucleus with a distinct thickening (arrow), providing evidence for the shape and orientation of the C-shaped rod apparatus.

The cells of *Bihospites bacati *n. gen. et sp. were elongated with a somewhat rounded posterior end and were 40-120 μm long and 15-30 μm wide (n = 200). The cells contained a brownish (or greenish) body near the posterior end of the cell and a variable number of distinctive black bodies at the anterior half of the cell (Figure 1A, B). The cells of *B. bacati *had two heterodynamic flagella that were inserted subapically within a depression. The longer anterior (dorsal) flagellum extended forward and continuously probed the substrate during 'gliding' movements (Figure 1B); periodically, the tip of the anterior flagellum would adhere to the substrate and abruptly drag the cell forward. The recurrent (posterior) flagellum was slightly longer than the cell body and trailed freely beneath the cell. The cells of *B. bacati *were plastic and capable of rhythmic deformations ranging from contracted to relaxed states that were reminiscent of "euglenoid movement" (Figure 1C). The cells divided from anterior to posterior along the longitudinal axis (Figure 1D). Cyst formation or sexual reproduction was not observed. Cells of *B. bacati *were found all year round, although the abundance of this species decreased significantly during the winter months.

### Cell Surface

**Figure 2:**
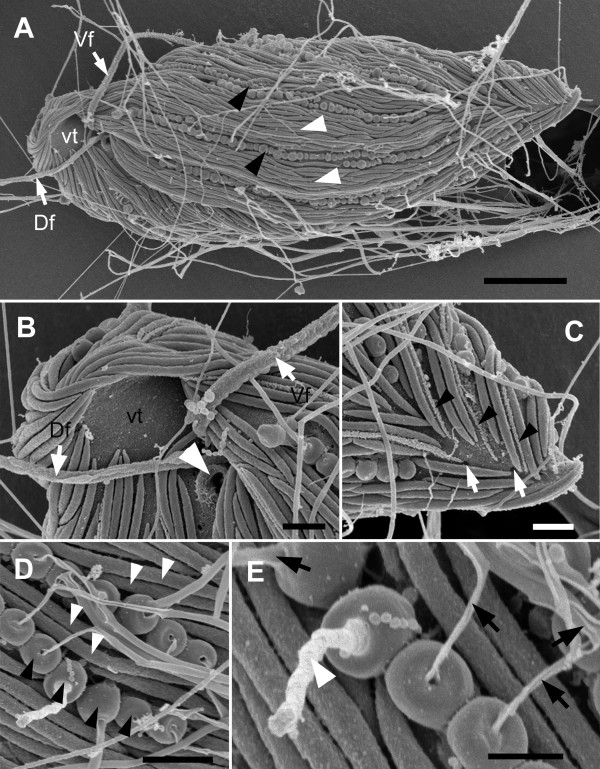
**Scanning electron micrographs (SEM) of *Bihospites bacati *n. gen. et sp**. **A**. Ventral view of *B. bacati *showing a cell covered with rod-shaped and spherical-shaped episymbiotic bacteria (white arrowheads and black arrowheads, respectively), the vestibulum (vt), dorsal flagellum (Df) and ventral flagellum (Vf) (bar = 15 μm). **B**. High magnification of the vestibular opening (vt), showing the open cytostome (white arrowhead), and the dorsal (Df) and ventral flagella (Vf) without flagellar hairs. **C**. High magnification SEM showing the posterior end of *B. bacati*, in ventral view, and the external appearance of the raised articulation zones between S-shaped folds in the host cell surface (black arrowheads). The white arrows show pores on the cell surface. **D**. High magnification SEM showing the rod-shaped (white arrowheads) and spherical-shaped episymbionts. **E**. High magnification SEM of the spherical-shaped episymbionts showing discharged threads (black arrows) through an apical pore (bar = 0.5 μm). The white arrow shows the initial stages of the ejection process. (**B**-**D **bar = 1 μm).

**Figure 3:**
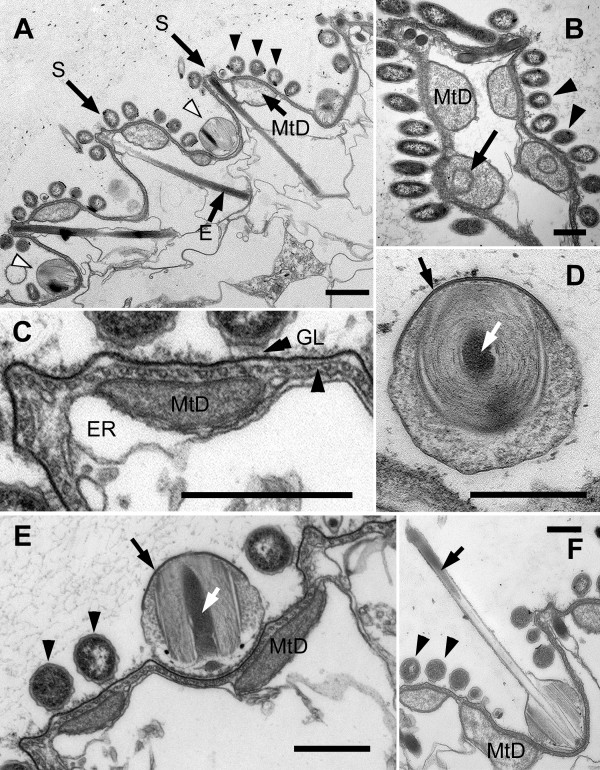
**Transmission electron micrographs (TEM) of the cell surface of *Bihospites bacati *n. gen. et sp**. **A**. Cross-section of cell showing a series of S-shaped folds in the cell surface. Elongated extrusomes (E) positioned beneath the raised articulation zones between the S-shaped folds (S). Cell surface covered with rod-shaped bacteria (black arrowheads), in cross section, and spherical-shaped bacteria (white arrowheads). Mitochondrion-derived organelles (MtD) underlie the cell surface. (bar = 1 μm). **B**. TEM showing mitochondrion-derived organelles (MtD) with zero to two cristae (arrow). Arrowheads show transverse profiles of rod-shaped episymbionts on cell surface. **C**. High magnification TEM of the host cell surface showing glycogalyx (GL) connecting episymbionts to plasma membrane. Plasma membrane subtended by a thick layer of glycoprotein (double arrowhead) and a continuous row of microtubules linked by short 'arms' (arrowhead). Mitochondrion-derived organelles (MtD) positioned between the row of microtubules and the endoplasmic reticulum (ER). **D**. Oblique TEM section of spherical-shaped episymbiont showing electron-dense apical operculum (black arrow) and the extrusive thread coiled around a densely stained core region (white arrow). **E**. High magnification TEM of cell surface showing mitochondrion-derived organelles (MtD), rod-shaped episymbionts (arrowheads), and spherical-shaped episymbiont (black arrow) sitting within a corresponding concavity in the host cell. Core region of the spherical-shaped episymbiont (white arrow) in longitudinal section. **F**. TEM of spherical-shaped episymbiont showing discharged extrusive thread (arrow). Electron-dense material corresponding to the core is positioned at the tip of the discharged thread (arrow). Arrowheads indicate rod-shaped bacteria on cell surface (**B**-**F **bar = 500 nm).

The cell surface of *B. bacati *was covered with two different morphotypes of episymbiotic bacteria: (1) more abundant rod shaped episymbionts and (2) spherical-shaped episymbionts (Figure 1E, 2). The rod-shaped episymbionts were 3-5 μm long and were arranged in bands, about 7 μm wide, along the longitudinal axis of the host cell (Figure 2A). These bands peeled off when the host cell deteriorated. The longitudinal bands of rod-shaped episymbionts were separated and defined by single or double rows of spherical episymbionts, each about 0.6 μm in diameter (Figure 2A-E). These longitudinal rows usually extended nearly the entire length of the host cell and were helically organized when the host cells were in a contracted state (Figure 1C, 2A). The rod-shaped episymbionts were connected to the plasma membrane of the host by a glycocalyx-like material (Figure 3A-E). The spherical-shaped episymbionts were attached to the host within a corresponding concavity in the host plasma membrane (Figure 3E). The spherical-shaped episymbionts were highly organized and possessed an extrusive apparatus consisting of an apical "operculum" and a tightly coiled internal thread around a densely stained core (Figure 3D-F). The coiled thread was capable of rapid discharge through an apical pore when disturbed during chemical fixation for electron microscopy (Figure 2A, D-E); the densely stained core was discharged first, and the coiled thread followed (Figure 3F).

The ultrastructure of the host cell surface, beneath the episymbionts, consisted of a plasma membrane that was organized into a repeated series of S-shaped folds (i.e., "strips") (Figure 1C, 3A), a thin layer of glycoprotein, and a corset of microtubules (Figure 3C). The longitudinal rows of spherical-shaped episymbionts were associated with the troughs of the S-shaped folds (Figure 3A). The raised articulation zones between the S-shaped folds were visible in (i) light micrographs of contracted cells (Figure 1C), (ii) scanning electron micrographs near the posterior end of the host cell (Figure 2C), and (iii) transmission electron micrographs (Figure 3A). The corset of microtubules beneath the folds formed a continuous row and was linked together by short "arms" (Figure 3C). Tubular cisternae of endoplasmic reticulum and a layer of double-membrane bound mitochondrion-derived organelles (MtD) were positioned immediately below the superficial corset of microtubules (Figure 3A-C, E-F). The mitochondrion-derived organelles contained a granular matrix and none or very few cristae per TEM profile (Figure 3B). There was no evidence of kinetoplast-like inclusions or any other kind of packed DNA within the matrix of the mitochondrion-derived organelles.

**Figure 4:**
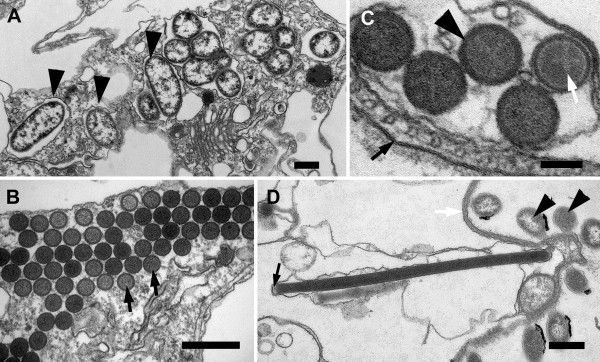
**Transmission electron micrographs (TEM) of *Bihospites bacati *n. gen. et sp. showing intracellular bacteria and extrusomes**. **A**. TEM showing a cell containing numerous intracellular bacteria (arrowheads) within vacuoles. **B**. Transverse TEM showing a battery of extrusomes (arrows) (**A**, **B**, bar = 500 nm). **C**. High magnification TEM of extrusomes showing a dense outer region (arrowhead) and a granular core containing a lighter cruciform structure (white arrow). Black arrow denotes the plasma membrane of the host (bar = 100 nm). **D**. TEM showing a longitudinal section of an extrusome; the proximal end is indicated with a black arrow. Arrowheads denote rod-shaped bacteria on the cell surface (bar = 500 nm).

The cytoplasm of the host cell was highly vacuolated and contained clusters of intracellular bacteria within vacuoles (Figure 4A). Batteries of tubular extrusomes, ranging from only a few to several dozen, were also present within the host cytoplasm (Figure 4B). The extrusomes were circular in cross-section and had a densely stained outer region that surrounded a lighter, granular core; a cruciform element was observed in cross-section of some extrusomes (Figure 4C). The extrusomes were approximately 4 μm long, and many of them were positioned immediately beneath the raised articulation zones between the S-shaped surface folds (Figure 3A, 4D).

### Nucleus, C-shaped Rod Apparatus, Cytostomal Funnel and Vestibulum

**Figure 5:**
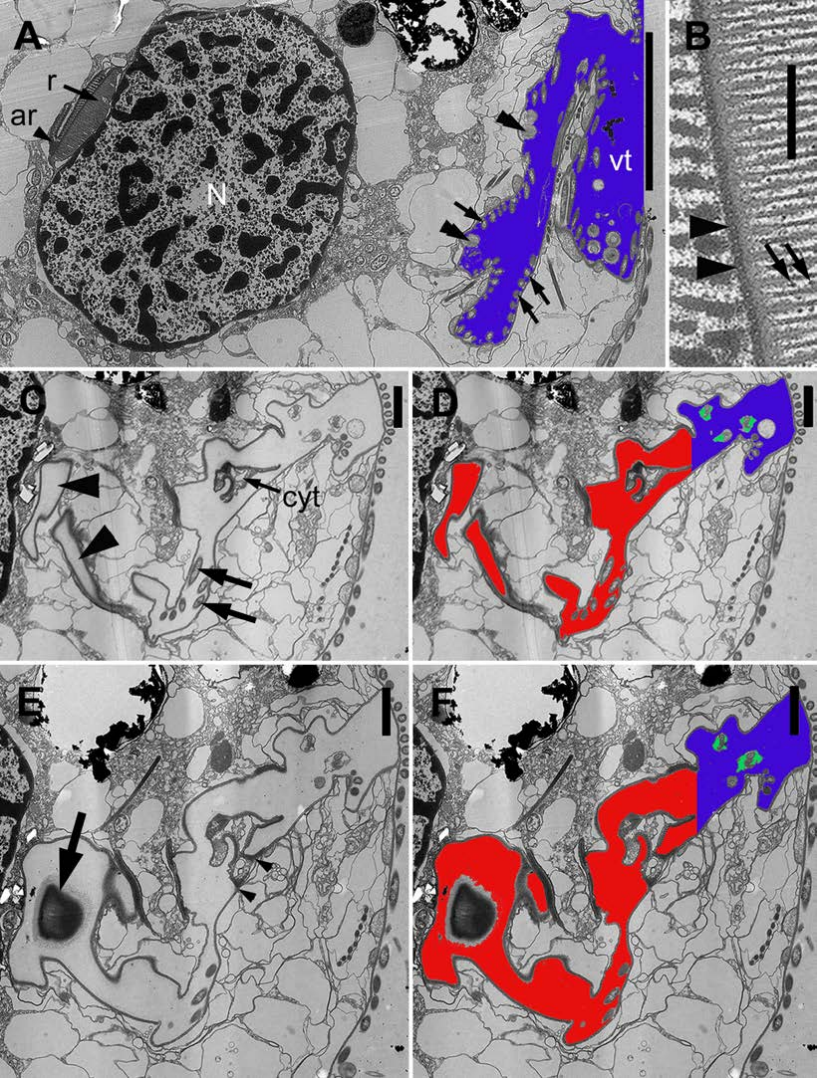
**Transmission electron micrographs (TEM) of non-consecutive serial sections of *Bihospites bacati *n. gen. et sp. through the vestibular region of the cell**. **A**. TEM showing the nucleus (N) with condensed chromatin, the dorsal side of the C-shaped rod apparatus consisting of the main rod (r) and the accessory rod (ar), and the vestibulum (vt). Several rod-shaped bacteria (black arrows) and spherical-shaped bacteria line inner surface of the vestibulum (vt) (bar = 10 μm). **B**. High magnification view of the C-shaped rod apparatus in Figure A showing the single row of microtubules (arrowheads) positioned at the junction between the tightly connected rod and accessory rod. Granular bodies (arrows) are present between the parallel lamellae that form the main rod (bar = 500 nm). **C, D**. Transverse TEMs showing the cytostomal funnel (cyt) and two separate lobes of the feeding pocket (arrowheads). Bacterial profiles can be seen inside the feeding pocket (arrows). Figure D uses color to distinguish between the feeding pocket (red), the vestibulum (blue), and the two branches of the flagellar pocket (green). **E, F**. Transverse TEMs at a more posterior level than in Figure C-D showing the posterior end of the main C-shaped rod (arrow) emerging within the posterior end of the feeding pocket. The cytostomal funnel (arrowheads) opens and fuses with the feeding pocket. Figure F uses color to distinguish between the feeding pocket (red), the vestibulum (blue), and the two branches of the flagellar pocket (green). (**C-F **bar = 2 μm).

**Figure 6:**
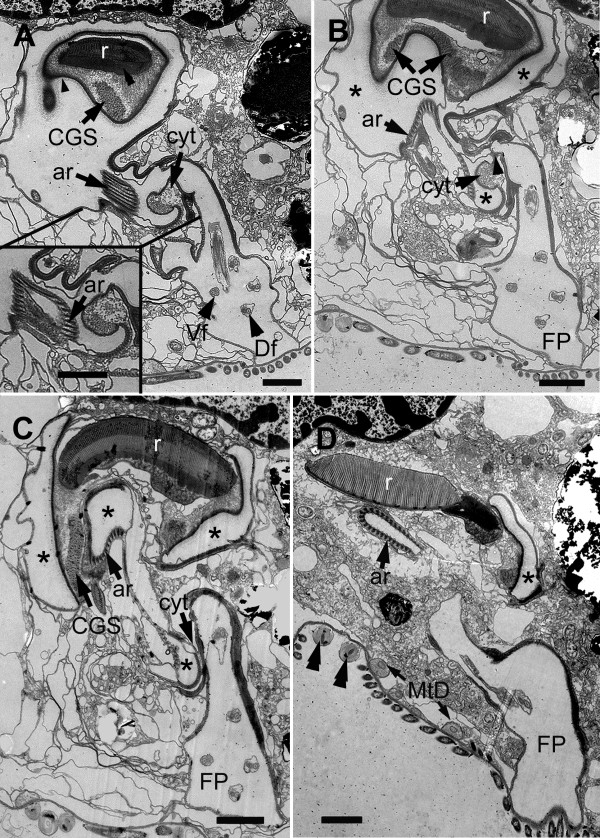
**Transmission electron micrographs (TEM) of non-consecutive serial sections through the flagellar apparatus and feeding pockets of *Bihospites bacati *n. gen. et sp**. TEMs taken at levels posterior to those shown in Figure 5 and presented from anterior (A) to posterior (D). **A**. TEM showing the posterior end of the main C-shaped rod (r) embedded in an amorphous matrix (double arrowhead) and surrounded by a thick membrane with fuzzy material (arrowhead). At this level, the rod is associated with 'congregated globular structure' (CGS), and the striated fibres that form the accessory rod (ar) appear near the cytostomal funnel (cyt) at the junction between the feeding pocket and the flagellar pocket. Inset: TEM showing the accessory rod (ar) in a subsequent posterior section, as it starts to open up. Vf = ventral flagellum; Df = dorsal lagellum. **B**. TEM showing the separation (arrowhead) of the feeding pocket (asterisks) from the flagellar pocket (FP) near cytostomal funnel (cyt) and the expanding accessory rod (ar). **C**. TEM showing the diminishing feeding pocket (asterisks), the cytostomal funnel (cyt), and the separate flagellar pocket (FP). **D**. TEM showing the accessory rod (ar) with its characteristically folded shape becoming more tightly linked to the main rod (r). Lobes of the feeding pocket (asterisk) and the flagellar pocket (FP) are also still visible. MtD = mitochondrion-derived organelle; double arrowheads = spherical-shaped episymbionts. (bars = 2 μm).

**Figure 7:**
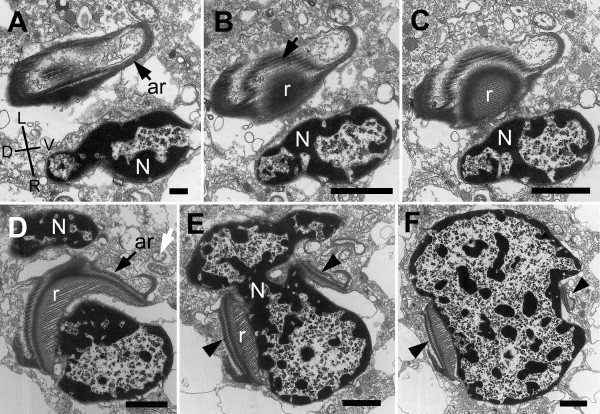
**Transmission electron micrographs (TEM) of non-consecutive serial sections through the anterior part of the nucleus of *Bihospites bacati *n. gen. et sp**. Figures 7A-F are presented from anterior to posterior. **A**. TEM showing the nucleus (N) and the accessory rod (ar) surrounded by electron-dense material (Images are viewed from the anterior side of the cell: D, dorsal; L, left side of the cell; R, right side of the cell; V, ventral). **B-C**. TEMs showing the main rod (r) near the striated fibres (SF) of the accessory rod (arrow). **D**. TEM showing the left side of the nucleus (N) appearing behind the rod (r) and accessory rod (ar). The white arrow shows the presence of bacteria near the rod. **E**. TEMs showing the main rod (r) and the accessory rod (arrowheads) on the dorsal and ventral sides of the nucleus. **F**. Transverse TEM at the level of the vestibulum showing the disappearance of the ventral side of the main rod (r) and the drastic reduction of the accessory rod (arrowhead). Note the indentations in the nucleus for accommodating the main rod and accessory rod (**A **bar = 500 nm; **B-F **bar = 2 μm).

**Figure 8:**
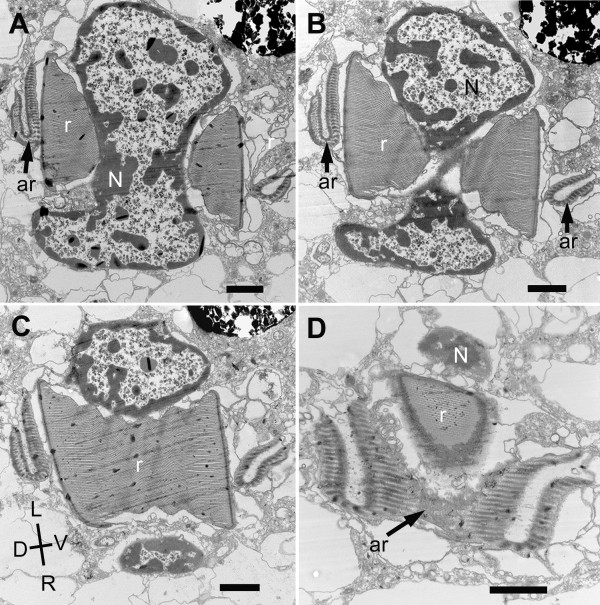
**Transmission electron micrographs (TEM) of non-consecutive serial sections through the posterior part of the nucleus of *Bihospites bacati *n. gen. et sp**. Figures 8A-D are presented from anterior to posterior. **A-C**. TEMs showing the rod (r) and the folded accessory rod (ar) nestled within indentations in the dorsal and ventral sides of the nucleus. The ventral part of the accessory rod runs close to the main rod for most of its length and extends toward the flagella on the ventral side of the cell. N = nucleus; D, dorsal; L, left side of the cell; R, right side of the cell; V, ventral; Images are viewed from the anterior side of the cell. **D**. TEMs showing the main rod (r) and the accessory rod (ar) reaching the posterior end of the nucleus (N). The main rod consists of parallel-arranged lamellae. Most of the nucleus and the main rod have disappeared from the section. The accessory rod (ar) consists of striated fibres that wrap around the main rod and the nucleus (bars = 2 μm).

**Figure 9:**
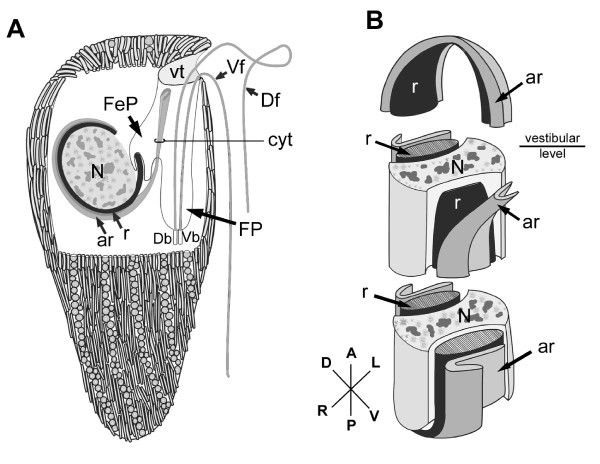
**Diagrams showing a reconstruction of the ultrastructure of *Bihospites bacati *n. gen. et sp**. Relationships between C-shaped rod apparatus, nucleus, cytostomal funnel, feeding pocket, flagellar pocket and vestibulum, as inferred from serial transmission electron microscopy (TEM), scanning electron microscopy (SEM), and light microscopy (LM). **A**. Cell viewed from the right side showing the positions of the nucleus (N), the C-shaped main rod (r), the accessory rod (ar), and the cytostomal funnel (cyt) in relation to the feeding pocket (FeP), the flagellar pocket (FP) and the vestibulum (vt); Vf = ventral flagellum; Df = dorsal flagellum; Db = dorsal basal body; Vb = ventral basal body. **B**. Diagram emphasizing the relationship between nucleus (N), main rod (r), and folded accessory rod (ar). The diagram is divided into three sections; and the nucleus removed from the top section for clarity. Posterior end of the main rod positioned at the level of the vestibulum on the ventral side of the nucleus. This rod extends posteriorly and then encircles the posterior, dorsal and anterior ends of the nucleus before terminating on the ventral side of the nucleus just above the vestibulum; therefore, this rod is C-shaped. The folded accessory rod runs along the C-shaped main rod for most of its length, terminating at the same point just above the vestibulum; however, on the ventral side of the nucleus, the posterior end of the accessory rod extends both anteriorly, defining the cytostomal funnel (cyt), and ventrally toward the ventral basal body.

The nucleus of *B. bacati *was positioned in the anterior half of the cell and had permanently condensed chromosomes (Figure 1A, 5A). The nucleus was also closely linked to a robust rod apparatus (Figure 1F). Serial sections through the entire nucleus demonstrated that a C-shaped system of rods formed a nearly complete ring around an indented nucleus (Figure 5A, 6, 7, 8 and 9). The C-shaped system of rods consisted of two main elements: (1) a main rod that was nestled against the indented nucleus (Figure 7, 8 and 9) and (2) a folded accessory rod that was pressed tightly against the outer side of the main rod for most of its length. We refer to this two-parted arrangement as the "C-shaped rod apparatus" (Figure 5A, 6, 7, 8 and 9). The main rod was composed of a dense cluster of parallel lamellae that often appeared corrugated, while the accessory rod was composed of striated fibres (SF) (Figure 5A-B, 6, 7 and 8). Granular bodies of approximately 35 nm in diameter were observed in the spaces between the parallel lamellae of the main rod (Figure 5B). The ventral side of the main rod was embedded in an amorphous matrix that became thinner toward the posterior end of the cell, until it disappeared altogether (Figure 6A-D). A single row of longitudinal microtubules lined the external side of the main rod, which delimited the boundary between the main rod and the accessory rod for most of their length (Figure 5A-B).

The anterior ends of both C-shaped rods terminated near the antero-ventral region of the nucleus (Figure 9). The posterior end of the main rod was positioned within the posterior region of a feeding pocket (Figure 5C-F, 9). This feeding pocket merged together with the flagellar pocket and formed a common subapical concavity in the cell or a "vestibulum" (Figure 2B, 5, 9A). A novel "cytostomal funnel" was positioned at the junction, and therefore demarcated the boundary, between the feeding pocket and the flagellar pocket (Figure 5, 6, 9A). The cytostomal funnel was an anterior extension of the posterior end of the accessory rod that eventually opened within the subapical vestibulum (Figure 2B, 5, 6 and 9A). Some microtubules associated with the posterior end of the accessory rod also extended toward the ventral side of the cell and appeared to become continuous with the (ventral flagellar root) microtubules that reinforced the flagellar pocket (not shown).

The posterior region of the feeding pocket also contained a "congregated globular structure" (CGS) that was associated with the posterior end of the main rod (Figure 6A-B). The posterior end of the folded accessory rod became more robust as the serial sections moved from the posterior end of the feeding pocket toward the posterior end of the cell (Figure 6, 9). The posterior end of the folded accessory rod was initially positioned between the feeding apparatus and the flagellar apparatus; the accessory rod then gradually became more robust and more tightly associated with the main rod as both of the rods migrated around the posterior side of the nucleus and toward the dorsal side of the nucleus (Figure 6A-D, 9). Moreover, as the sections continued posteriorly, the feeding pocket and the CGS that surrounded the main rod diminished, and ultimately only the main rod and the accessory rod remained (Figure 6C-D).

Serial sections through the anterior region of the nucleus, moving from anterior to posterior, demonstrated the C-shaped curvature of the rod apparatus (Figure 7, 9). These sections also demonstrated how the anterior ends of both the main rod and the accessory rod terminate on the ventral side of the indented nucleus near the vestibulum (Figure 7F). Similarly, serial sections through the posterior region of the nucleus, moving from anterior to posterior, demonstrated the C-shaped curvature of the rod apparatus and its relationship to the indented nucleus (Figure 8, 9).

### Flagellar Root System

**Figure 10:**
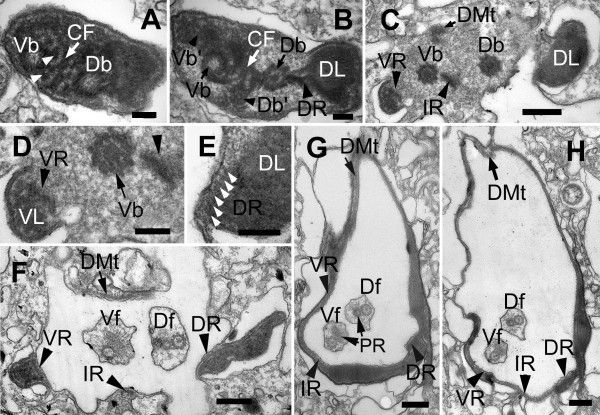
**TEM micrographs showing sections of basal bodies, flagellar roots and associated structures, of *Bihospites bacati *n. gen. et sp**. **A-H **from proximal to distal end of flagellar pocket. **A**-**C**. Non-consecutive serial sections showing origin and organization of flagellar pocket. **A**. High magnification TEM of proximal region of basal bodies showing dorsal and ventral basal bodies (Db and Vb) linked by a connecting fibre (CF). Basal bodies with cartwheel structures associated to electron-dense fibres (arrowheads). **B**. TEM showing accessory dorsal and ventral basal bodies (Db' and Vb') on the left of the two main basal bodies. Dorsal root (DR) connects to electron-dense body (dorsal lamella=DL), on right side of Db. **C**. TEM showing intermediate root (IR) associated with right side of Vb. Ventral root (VR) associated with electron-dense material that becomes ventral lamella (VL). Row of dorsal microtubules (DMt), not associated with basal bodies. **D**. Detail of ventral side of Figure C showing Vb, VR formed by four microtubules, VL and intermediate root (arrowhead), initially composed of eight microtubules. **E**. Detail of dorsal side of Figure C showing DR, with six microtubules (white arrowheads), and DL. **F**. TEM showing three flagellar roots and DMt around flagellar pocket. Df = dorsal flagellum; Vf = ventral flagellum. **G-H**. Non-consecutive serial TEM sections of flagellar pocket showing Df and Vf with paraxial rods (PR), flagellar roots, DMt of microtubules lining flagellar pocket, and DL and VL. (**A-B **and **D-E **bars = 200 nm; **C **and **F **bars = 500 nm; **G-H **bars = 2 μm)

**Figure 11:**
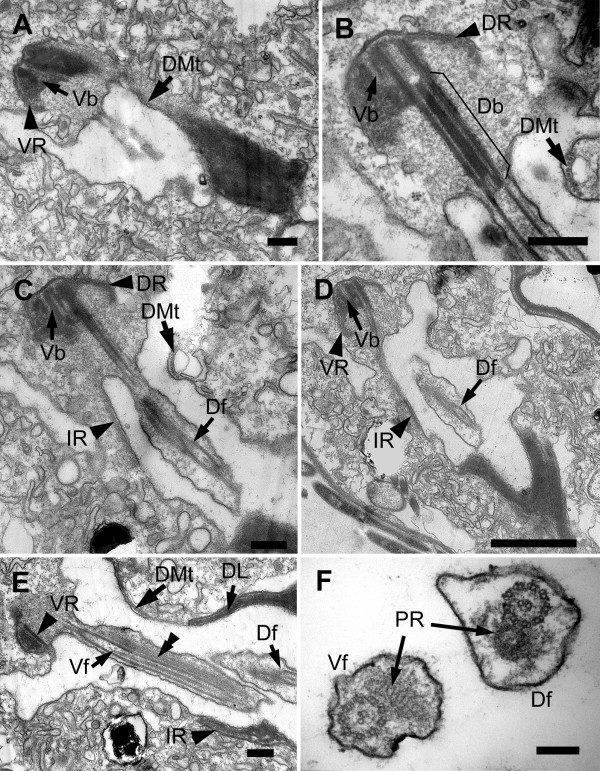
**Transmission electron micrographs (TEM) of *Bihospites bacati *n. gen. et sp. showing the emergence and organization of the flagella**. **A**. Longitudinal TEM through the electron-dense region near the origin of the basal bodies. The ventral root (VR) originates from the ventral basal body (Vb). A row of microtubules (DMt) lines the dorsal side of the incipient flagellar pocket. **B**. Longitudinal TEM through the dorsal flagellum showing the dorsal basal body (Db) associated with the dorsal flagellar root (DR), the ventral basal body (Vb), and the dorsal microtubules (DMt). **C-D**. TEM sections showing the dorsal flagellum (Df) and the intermediate root (IR) associated with the ventral basal body (Vb). **E**. TEM showing oblique sections through both flagella and the positions of the VR, IR and DMt in the flagellar pocket. The electron-dense material from which the flagellar apparatus originated in Figure A elongates to form the dorsal lamella (DL). The double arrowheads show the paraxial rod in the ventral flagellum (Vf). **F**. Transverse TEM of the Df and Vf showing the 9+2 arrangement of microtubules in the axoneme and the heteromorphic paraxial rods (PR). (**A-E **bars = 500 nm; **F **bar = 200 nm)

Two flagella emerged from the base of the flagellar pocket (Figure 2A-B, 10A-F, 11A-E). Each flagellum had a paraxial rod (PR) in addition to the 9+2 arrangement of microtubules forming the axoneme (Figure 10G-H, 11F). The PR in the dorsal flagellum (Df) had a whorled disposition, whereas the PR of the ventral flagellum (Vf) had a lattice-like arrangement of parallel fibres (Figure 11F). No mastigonemes were observed on either flagellum (Figure 2A-B). The dorsal basal body contained a long opaque core (Figure 11B). Both basal bodies were approximately 1.7 μm long and were linked by a connecting fibre (CF) (Figure 10A-B). A cartwheel structure was present at the proximal end of both basal bodies (Figure 10A-B). Two accessory basal bodies (Db' and Vb') were observed on the ventral side of the Db and the dorsal side of the Vb (Figure 10B).

The flagellar root system is described here from the proximal to the distal end of the basal bodies as viewed from the anterior end of the cell. The basal bodies were associated with three asymmetrically arranged flagellar roots. A dorsal root (DR) originated from the dorsal-right side of the Db (Figure 10B, 11B-C) and was formed of approximately six microtubules (Figure 10E). A ventral root (VR) connected to the dorsal-right side of the ventral basal body (Figure 11A, D-E) and was comprised initially of four microtubules (Figure 10D). An intermediate root (IR), originally formed of about eight microtubules (Figure 10F), emerged from the left side of the Vb (Figure 10C-D). The ventral root and the intermediate roots ultimately fused, forming a continuous VR-IR row of microtubules around the flagellar pocket (Figure 10G-H). A band of dorsal microtubules (DMt), not directly associated to the basal bodies, lined the dorsal side of the flagellar pocket (Figure 10C, F; 11A-E). Toward the anterior end of the cell, the number of microtubules increased one by one, until the band reached the dorsal root (DR). The DMt and the DR eventually fused and formed a single band of microtubules around the flagellar pocket (Figure 10G-H).

The DR and VR were associated with two electron dense bodies that elongated to form a dorsal lamina (DL) and a ventral lamina (VL), respectively (Figure 10C-H). Both laminae extended anteriorly and ended up reinforcing the walls of the flagellar pocket (Figure 10G-H). The DR, together with the DL, supported the dorsal-left side of the pocket, and the DMt supported the dorsal-right side. The VR - reinforced by the VL - lined the ventral side of the pocket and was in contact with the IR that lined the ventral-left side of the flagellar pocket. The microtubules of the DMt and the VR became part of the elements forming the cytostomal funnel and accessory rod (i.e., the C-shape rod apparatus in general), and both the DR and the IR became part of the sheet of microtubules underlining the plasma membrane of the entire cell.

### Molecular Phylogenetic Position

**Figure 12:**
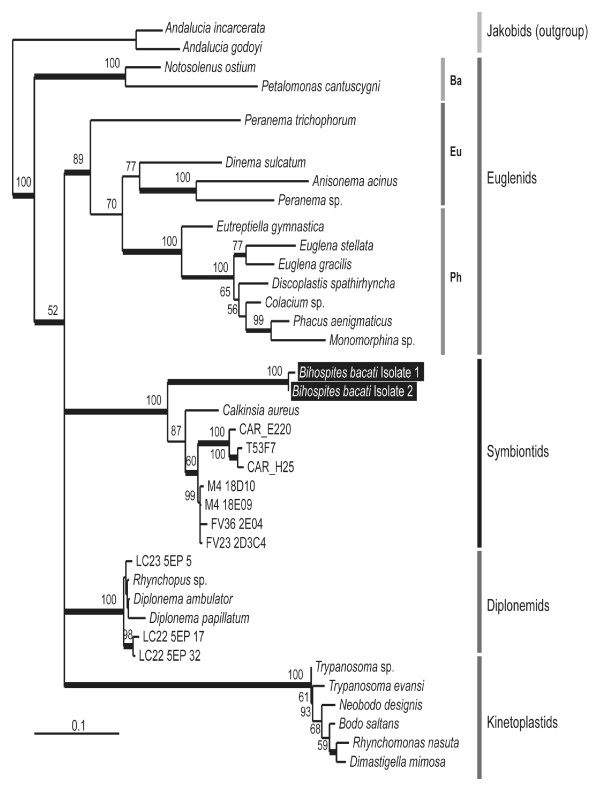
**Phylogenetic position of *Bihospites bacati *n. gen. et sp. within the Euglenozoa as inferred from small subunit (SSU) rDNA sequences**. Maximum likelihood (ML) analysis of 35 euglenozoan taxa, rooted with two jakobids (*Andalucia incarcerata *and *A. godoyi*). Only ML boostraps greater then 50% are shown. Thick branches correspond to Bayesian posterior probabilities over 0.95. Ba, bacterivorous taxa; Eu, eukaryovorous taxa; Ph, photosynthetic taxa.

In order to infer the phylogenetic position of *B. bacati*, we PCR-amplified and sequenced the nearly complete SSU rDNA gene (2057 bp) from two independent isolates. The sequences contained expansions typical of euglenozoan SSU rDNA genes. First, we carried out a 40-taxon Maximum likelihood (ML) analysis that included sequences representing all of the major groups of eukaryotes; the resulting phylogeny showed *B. bacati *grouped strongly within the Euglenozoa (not shown). A second analysis included 37 taxa representing all of the major lineages of euglenozoans. The phylogenetic analyses showed that the euglenozoan sequences clustered in five main subgroups with high statistical support (Figure 12): (i) a kinetoplastid clade, (ii) a diplonemid clade, (iii) a bacteriovorous euglenid clade, (iv) a eukaryovorous + phototrophic euglenid clade and (v) the Symbiontida, a newly named clade that includes *Calkinsia aureus *and several environmental sequences. *Bihospites bacati *clustered with the Symbiontida with extremely high statistical support (ML bootstrap value = 100% and Bayesian posterior probability > 0.95), as the sister lineage to the rest of this group. *Calkinsia aureus *branched next within the Symbiontida and formed the sister lineage to several environmental sequences (Figure 12). However, the relationship of the Symbiontida to the other main subgroups within the Euglenozoa was unclear.

## Discussion

*Bihospites bacati *n. gen et sp. possesses all three synapomorphies that unify the Euglenozoa: a tripartite flagellar root system, heteromorphic paraxial rods and tubular extrusomes. Concordantly, our analyses of SSU rDNA sequences clearly places *B. bacati *within the Euglenozoa, specifically within the Symbiontida. Several studies based on environmental sequences indicated the existence of a novel rDNA clade of euglenozoans [9-11]. However, the Symbiontida was proposed after the ultrastructural description and molecular phylogeny of *C. aureus *strongly grouped this species with these environmental sequences, as a distinct subgroup within the Euglenozoa [19]. Nonetheless, it was not clear in that study whether the Symbiontida was a new clade of euglenozoans or a subclade within one of the three previously recognized members of the Euglenozoa (i.e., kinetoplastids, diplonemids and euglenids). Our comprehensive characterization of *B. bacati *sheds considerable light onto this question.

### Remnants of Pellicle Strips

*Bihospites bacati *possesses a cell surface consisting of S-shaped folds, microtubules and endoplasmic reticulum that is similar to the pellicle of S-shaped strips found in euglenids. In most photosynthetic euglenids, the pellicle strips usually consist of a robust proteinaceous frame that supports and maintains the shape of the cell, even during euglenoid movement [21-23]. However, like in most phagotrophic euglenids, there is no robust proteinaceous frame in *B. bacati*. Articulation zones between strips in the euglenid pellicle function as 'slipping points' around which the pellicle can change shape rather freely; moreover, the relative number of strips in each euglenid species reflects phylogenetic relationships and the degree of cell plasticity [24]. Due to the extreme flexibility of the cell surface in *B. bacati*, it was not possible to determine an exact number of S-shaped folds in the cell surface. Nonetheless, the microtubular corset in most euglenids is regularly interrupted, thus forming groups of a few microtubules associated with each pellicle strip, the number of which varies between species [21-23]. By contrast, the microtubules beneath the plasma membrane in *B. bacati *form a continuous corset over the entire cell, much like that found in several phagotrophic euglenids (e.g., *Dinema *[21]) and in symbiontids (*C. aureus *[19] and *Postgaardi mariagerensis *[16]).

### A Novel Feeding Apparatus Consisting of Rods

*Bihospites bacati *possesses a well-developed C-shaped rod apparatus consisting of a main rod and an associated accessory rod. Several heterotrophic euglenids [25-30], and some species of diplonemids [31-36], have been described with feeding apparatuses consisting of two main rods; some species also have corresponding accessory rods (e.g. *Peranema trichophorum *has two main rods and two folded accessory rods) or have a branched rod that gives the appearance of three main rods (e.g., *Entosiphon*). Nonetheless, there are several differences between these rods and those described here for *B. bacati*. Firstly, *B. bacati *only has one main rod and one folded accessory rod; this configuration has never been described so far. Secondly, the vast majority of this apparatus tightly encircles the nucleus in a C-shaped fashion, the functional significance of which is totally unclear. The straight rods in euglenids support and line a conspicuous feeding pocket, whereas the feeding pocket in *B*. *bacati *only associates with the posterior end(s) of the C-shaped rod apparatus. Thirdly, the main C-shaped rod in *B. bacati *is formed by a highly novel arrangement of tightly packed lamellae, and only a single row of microtubules originating from the VR separates the main C-shaped rod from the folded accessory rod. This row of microtubules demarcates the end of each lamella in the main rod. In all of the previously described euglenozoan species, different rods are formed by different proportions of amorphous material (not parallel lamellae) and microtubules originating from the ventral root of the ventral basal body. Fourthly, the posterior terminus of the accessory rod in *B. bacati *participates in the formation of a novel cytostomal funnel that extends anteriorly and merges with the subapical vestibulum. The cytostomal funnel presumably closes the connection between the flagellar pocket and the vestibulum during feeding. Although the cytostomal funnel in *B. bacati *is likely homologous to the "vanes" described in several different phagotrophic euglenids, the unusual ultrastructural features of *B. bacati *made this inference somewhat tenuous. Nonetheless, the additional "congregated globular structure" (CGS) at the posterior end of the main rod in *B. bacati *is also present in *Calkinsia aureus *[19]. However, the feeding apparatus in *C. aureus *lacks conspicuous rods (or vanes) and consists mainly of a feeding pocket reinforced by microtubules from the VR, similar to the MTR pockets of other euglenozoans (e.g., *Petalomonas*). Overall, the C-shaped rod apparatus in *B*. *bacati *appears to contain some homologous subcomponents with phagotrophic euglenozoans (e.g., a main rod and a folded accessory rod), but, as highlighted above, this apparatus is novel in most respects.

The presence of a highly plastic cell surface, an elaborate feeding apparatus, and brownish bodies, reminiscent of food vacuoles, suggests that *B. bacati *is capable of engulfing large prey cells such as other eukaryotes [1,3,24,27,29,37]; however, this species was never directly observed preying on (relatively large) microeukaryotic cells present in the environment. Nonetheless, the presence of intracellular bacteria surrounded by vacuoles near the feeding pocket indicates that *B. bacati *actively feeds on bacteria. It is also possible that *B. bacati *feeds on the rod shaped episymbiotic bacteria that grow over the host surface and into the subapical vestibulum.

### Extrusomes

Tubular extrusomes are present in several members of the Euglenozoa [16,19,36] and constitute a synapomorphy for the group. Among the Symbiontida, *C. aureus *has tubular extrusomes clustered in a single large battery that is longitudinally arranged and anchored to a novel "extrusomal pocket" [19]. Although *Bihospites bacati *also possesses tubular extrusomes, these organelles are not organized as a single battery. The extrusomes in *B. bacati *are arranged in several smaller clusters that are distributed in different places throughout the superficial cytoplasm; solitary extrusomes are organized consecutively beneath the articulation zones of the S-shaped pellicular folds or "strips". A similar arrangement of tubular extrusomes has also been observed in *P. mariagerensis *[16].

### Episymbiotic Bacteria

Several distantly related species of euglenozoans have been described with episymbiotic bacteria. These euglenozoans are usually phagotrophs that live in oxygen-depletd to anoxic marine environments, such as that in which *B. bacati *thrives [15,16,18,19,38,39]. However, two species of euglenids living in well-oxygenated, freshwater environments have also been described as having episymbiotic bacteria: the phototroph *Euglena helicoideus *[40], and the phagotroph *Dylakosoma pelophilum *[41]. The episymbionts so far encountered in euglenozoans are either rod-shaped (in *Euglena helicoideus *[40], *Postgaardi mariagerensis *[16], *Calkinsia aureus *[19,38]) or spherical-shaped (*D. pelophilum *[41]). *Bihospites bacati*, however, is the first euglenozoan described with both morphotypes of episymbionts.

Hypotheses about the role of rod-shaped bacteria in symbiotic relationships with eukaryotic hosts usually emphasize commensalism, where the bacteria benefit from metabolic byproducts secreted by the host [15,16,20]. It has also been proposed that the rod-shaped bacteria are chemoautotrophic sulphur or methanogenic-oxydizers and form a mutualistic relationship with the host [18], whereby the host provides anchorage for the bacteria and the bacteria detoxify the immediate environment for the host [39,42]. The episymbiotic bacteria may also serve as a food-source for the host, as has been observed in one ciliate [43].

Spherical episymbiotic bacteria have been reported in one other euglenozoan based only on light microscopy: the freshwater euglenid *D. pelophilum *[41]. However, this species has so far been poorly described and morphological characteristics of the bacteria are very difficult to evaluate; it was reported that the bacteria on the surface of *D. pelophilum *are 2 μm in diameter, twice the size of those in *B. bacati*. Spherical episymbiotic bacteria that are nearly identical at the ultrastructural level to those we describe here on *B. bacati *have been demonstrated on one species of hypotrich ciliate isolated from tidal pools [43-46]. Molecular phylogenetic evidence demonstrates that these episymbionts, called "epixenosomes", are novel lineages of verrucomicrobial bacteria, and experiments indicate that the extrusive nature of the spherical episymbionts function in defense against predators [43,45,46]. Therefore, these episymbionts improve the comparative context for understanding the origin(s) of different types of extrusive organelles in different lineages of eukaryotes (e.g., ejectosomes in cryptophytes and nematocysts in cnidarians and dinoflagellates). A more comprehensive examination and discussion of the biology and origins of the epixenosomes in *B. bacati *have been incorporated into a companion paper currently in preparation for publication (Breglia, Yubuki and Leander, unpubl. data).

### The Identity and Composition of the Symbiontida

Molecular phylogenetic analyses using SSU sequences place *B. bacati *as the earliest diverging branch within the Symbiontida. The Symbiontida are anaerobic and microaerophilic euglenozoans covered with rod-shaped bacteria that are in close association with a superficial layer of mitochondrion-derived organelles with reduced or absent cristae; accordingly, it was predicted that rod-shaped episymbionts are present in most (if not all) members of the group [19]. The morphology of *B. bacati *is concordant with this description, reinforcing the interpretation that the presence of episymbiotic bacteria is a shared derived character of the most recent ancestor of the Symbiontida. This hypothesis is more robustly corroborated when we consider that *B. bacati *and *C. aureus *form a paraphyletic assemblage near the origin of the Symbiontida. In other words, episymbiotic bacteria are no longer a character known only in a single lineage within this group. Given this context, current ultrastructural data indicate that *P. mariagerensis *is also a member of the Symbiontida (e.g., *B. bacati, C. aureus *and *P. mariagerensis *all lack flagellar hairs and possess rod-shaped episymbionts, a continuous corset of cortical microtubules, and a superficial layer of mitochondrion-derived organelles) [16,19]. This inference, however, needs to be examined more carefully with an ultrastructural characterization of the flagellar apparatus and feeding apparatus in *P. mariagerensis *and with molecular phylogenetic data from the host and the episymbionts.

The presence of episymbiotic bacteria and the superficial distribution of mitochondria with reduced cristae in *B. bacati, C. aureus *and *P. mariagerensis *indicate a mutualistic relationship that enabled both lineages to diversify within low-oxygen environments. Determining whether the episymbionts on *B. bacati*, *C. aureus *and other symbiontids are closely related will more robustly establish the identity and composition of the clade and potentially reveal co-evolutionary patterns between the symbionts and the hosts. The geographic distribution of *C. aureus *and *B. bacati *(i.e. seafloor sediments of Santa Barbara Basin, California and coastal sediments of British Columbia, Canada) suggests that the Symbiontida is more widespread and diverse than currently known. This view is supported by the existence of related environmental sequences originating from Venezuela, Denmark and Norway [9,11,13]. Moreover, an organism with striking morphological resemblance to *B. bacati *has been previously observed in the Wadden Sea, Germany, [47]. More comprehensive sampling of anoxic and low-oxygen sediments around the world will shed considerable light on the abundances and ecological significance of this enigmatic group of euglenozoans.

## Conclusions

We described and characterized a novel flagellate from micro-aerobic marine sand: *Bihospites bacati *n. gen. et sp. Both comparative ultrastructure and molecular phylogenetic analyses strongly support the placement of *B. bacati *with the Euglenozoa and, more specifically, as a new member of the Symbiontida. An early diverging position of *B. bacati *within the Symbiontida is consistent with the presence of morphological features that are transitional between those found in *C. aureus *and phagotrophic euglenids: (1) a cell surface with strip-like S-shaped folds but lacking the proteinaceous frames of the euglenid pellicle, (2) a compact but robust rod-based feeding apparatus, and (3) a dense community of rod-shaped episymbiotic bacteria on the cell surface but without the elaborate extracellular matrix of *C. aureus*. Therefore, the molecular phylogenetic position and suite of intermediate ultrastructural features in *B. bacati *suggest that the most recent ancestor of the Symbiontida descended from phagotrophic euglenids. Although the close association of rod-shaped episymbiotic bacteria with the underlying mitochondria is a shared feature of symbiontids, the presence of extrusive verrucomicrobial episymbionts in *B. bacati *is highly unusual. These rapid-firing episymbionts could provide critical context for understanding the origin(s) of several different types of extrusive organelles in eukaryotes, and their discovery on this novel euglenozoan lineage underscores how little we know about the diverse symbiotic communities present in marine benthic environments.

## Methods

### Collection of organisms

Sediment samples were collected at low tide from the shoreline of Centennial Beach (Boundary Bay) in South-western British Columbia, Canada (49° 00' 4797''N, 123° 02' 1812''W), during the spring and summer of 2007 and 2008. The samples were taken at a depth of 1-3 cm below the sediment surface, from a conspicuous layer of black sand. The sediment samples were stored in flat containers at room temperature before individually isolated cells were prepared for light microscopy, electron microscopy and DNA extraction. Cells were extracted from the sand samples through a 48-μm mesh using the Uhlig melted seawater-ice method [48].

Attempts to culture the organism were made using two different media: ATCC 1728 (for growing *Isonema*) and CCAP 1259/1 (for growing *Petalomonas cantuscygni*). Both media were diluted in sterile seawater and kept under low oxygen conditions (oxygen content below 1%) using the ANAEROGEN™ COMPACT Kit system for anaerobic incubation; however, the cells did not reproduce and disappeared within 24 hours.

### Light and electron microscopy

Differential interference contrast (DIC) light micrographs were taken using a Zeiss Axioplan 2 imaging microscope and a Leica DC500 digital chilled CCD camera.

Cells isolated from the British Columbia locality were fixed for scanning electron microscopy (SEM) using the 4% osmium tetroxide vapour protocol described previously [1]. The cells were then transferred onto a 10-μm polycarbonate membrane filter, dehydrated with a graded ethanol series, and critical point dried with CO_2 _using a Tousimis Critical Point Dryer. The filter was then mounted on an aluminium stub, sputter coated with gold/palladium using a Cressington 208 HR High Resolution Sputter Coater, and observed with a Hitachi S-4700 field emission scanning electron microscope.

Cells isolated from the surrounding sediment were pre-fixed for transmission electron microscopy (TEM) using 4% (v/v) glutaraldehyde in 0.2 M sodium cacodylate buffer (SCB) (pH 7.2) with the addition of 0.3 M sorbitol. The pre-fixed cells were washed in 0.2 M SCB (pH 7.2) three times and embedded in 2% of low melting temperature agarose and post-fixed in 1% (w/v) osmium tetroxide in 0.2 M SCB (pH 7.2) at room temperature for 1 hr, before being dehydrated through a graded series of ethanol and 100% acetone. The dehydrated cells were then infiltrated with acetone-Epon 812 resin mixtures and 100% Epon 812 resin. Ultra-thin serial sections were collected on copper Formvar-coated slot grids, stained with 2% (w/v) uranyl acetate and lead citrate, and observed using a Hitachi H7600 electron microscope.

### DNA extraction, PCR amplification, alignment and phylogenetic analysis

Genomic DNA was extracted using the MasterPure Complete DNA and RNA purification Kit (Epicentre, WI, USA) from 30 cells that were individually isolated and washed three times in sterile seawater (i.e., "isolate 1"). This procedure was repeated three months later on a different sample of 30 individually isolated cells (i.e., "isolate 2"). Polymerase chain reactions (PCR) were performed using PuRe Taq Ready-To-Go PCR beads kit (GE Healthcare, Buckinghamshire, UK). Nearly the entire eukaryotic SSU rDNA gene was amplified from each isolate using the eukaryotic universal primers 5'- TGATCCTTCTGCAGGTTCACCTAC-3' [49] and 5'-GCGCTACCTGGTTGATCCTGCCAGT-3' [50]. PCR amplifications consisted of an initial denaturing period (95°C for 3 min), 35 cycles of denaturing (93°C for 45 s), annealing (5 cycles at 45°C and 30 cycles at 55°C, for 45 s), extension (72°C for 2 min), and a final extension period (72°C for 5 min). The amplified DNA fragments were purified from agarose gels using UltraClean 15 DNA Purification Kit (MO Bio, CA, USA), and subsequently cloned into the TOPO TA Cloning Kit (Invitrogen, CA, USA). Two clones of the eukaryotic SSU rRNA gene, from each of the two isolates (i.e., four clones in total), were sequenced with the ABI Big-Dye reaction mix using the vector primers and internal primers oriented in both directions. The new sequences were screened with BLAST, identified by molecular phylogenetic analysis, and deposited into GenBank: HM004353, HM004354.

The SSU rDNA sequences from *B. bacati *were analyzed within the context of two alignments: (1) a 40-taxon alignment consisting of taxa representing all of the major groups of eukaryotes (988 unambiguously aligned sites) and (2) a 37-taxon alignment consisting of taxa representing all of the major lineages of euglenozoans (760 unambiguously aligned sites). Ambiguously aligned positions and gaps were excluded from both analyses. Phylogenetic relationships were inferred using maximum likelihood (ML) and Bayesian methods with the programs PhyML [51] and MrBayes [52], respectively. For ML, the nucleotide datasets were analysed using a general-time-reversible (GTR) model of base substitutions, plus a gamma correction with eight substitution rate categories and the proportion of invariable sites (GTR + I + G). ML bootstrap analysis of 500 replicates was performed with the same parameters described above. For Bayesian analyses, the program MrBayes was set to operate with a gamma correction with eight categories and proportion of invariable sites, and four Monte-Carlo-Markov chains (MCMC) (default temperature = 0.2). A total of 2,000,000 generations was calculated with trees sampled every 50 generations and with a prior burn-in of 100,000 generations (i.e., 2,000 sampled trees were discarded). A majority rule consensus tree was constructed from 18,000 post-burn-in trees with PAUP* 4.0. Posterior probabilities correspond to the frequency at which a given node is found in the post-burn-in trees.

### Archiving

A digital archive of this paper is available from PubMed Central and print copies are available from libraries in the following five museums: Natural History Museum Library (Cromwell Road, London, SW7 5BD, UK), American Museum of Natural History (Department of Library Services, Central Park West at 79th St., New York, NY, 10024, USA), Muséum national d'Histoire naturelle (Direction des bibliothèques et de la documentation, 38 rue Geoffroy Saint-Hilaire, 75005 Paris, France), Russian Academy of Sciences (Library for Natural Sciences of the RAS Znamenka str., 11, Moscow, Russia) and Academia Sinica (Life Science Library, 128 Sec. 2 Academia Rd, Nankang Taipei 115, Taiwan R.O.C.).

### Formal Taxonomic Descriptions

Euglenozoa, Cavalier-Smith, 1981 [53]

Symbiontida, Yubuki, Edgcomb, Bernhard & Leander, 2009 [19]

***Bihospites *n. gen**. Breglia, Yubuki, Hoppenrath and Leander 2010

### Description

Uninucleate biflagellates; two heterodynamic flagella inserted subapically, with paraxial rods and no mastigonemes; flagella of approximately the cell length; elongated cells with a rounded posterior end; nucleus at anterior end of cell; cell covered with epibiotic bacteria of two different types: rod-shaped and spherical-shaped; cell surface with S-shaped folds; tubular extrusomes with cruciform core; presence of black bodies mainly at the anterior end of cell; rhythmic cell deformations and gliding motility.

### Type species

*Bihospites bacati*.

### Etymology

Latin *Bihospites*, with two guests. The generic name reflects the presence of two different episymbiont morphotypes: rod-shaped, and spherical-shaped episymbionts.

***Bihospites bacati *n. sp**. Breglia, Yubuki, Hoppenrath and Leander 2010

### Description

Cell elongated with rounded ends; cell size 40-120 μm in length and 15-30 μm in width; two heterodynamic flagella inserted subapically; anterior nucleus; cell covered with epibiotic bacteria of two different types: rod-shaped and spherical-shaped; cell surface with S-shaped folds; mitochondrion-derived organelles with reduced or absent cristae; feeding apparatus with conspicuous C-shaped rod and accessory rod that encircles the indented nucleus; the rod is formed by tightly packed, parallel-arranged lamellae; presence of black bodies, mainly at the anterior end of the cell; rhythmic cell deformations and gliding motility. Small subunit rRNA gene sequences [GenBank: HM004353, HM004354].

### Hapantotype

Both resin-embedded cells used for TEM and cells on gold sputter-coated SEM stubs have been deposited in the Beaty Biodiversity Research Centre (Marine Invertebrate Collection) at the University of British Columbia, Vancouver, Canada.

### Iconotypes

Figs 1A, 2A and 9A.

### Type locality

Tidal sand-flat at Centennial Beach, Vancouver, British Columbia, Canada (49°00' 4797''N, 123°02'1812''W).

### Habitat

Marine sand, black layer 2-3 cm deep.

### Etymology

Specific epithet, Latin *bacati*, ornamented with pearls. The etymology for the specific epithet reflects the presence of distinct longitudinal rows of spherical-shaped episymbionts, reminiscent of pearl necklaces.

### Registration of new genus and species name in ZooBank

LSID for article: urn:lsid:zoobank.org:pub:40211D82-B95C-494A-B8D0-7E061E80DD18

LSID for the genus *Bihospites*: urn:lsid:zoobank.org:act:794D6C7B-BFB1-45C7-8DDA-32D44F3B0E50

LSID for the species *B. bacati*: urn:lsid:zoobank.org:act:E1549565-5434-4F85-B936-7D0C485596B8

## Abbreviations

ar: accessory rod; CGS: congregated globule structure; Cyt: cytostome; Db: dorsal basal body; Db': dorsal pro-basal body; Df: dorsal flagellum; DL: dorsal lamina; DMt: dorsal microtubules; DR: dorsal root; E: extrusome; epi: epixenosome; ER: endoplasmic reticulum; FP: flagellar pocket; IR: intermediate root; LM: light microscope; MtD: mitochondrion-derived organelle; N: nucleus; Nu: nucleolus; PR: paraxial rod; r: rod; S: strips; SF: striated fibre; SEM: scanning electron microscope; TEM: transmission electron microscope; tz: transition zone; Vb: ventral basal body; Vb': ventral pro-basal-body; Vf: ventral flagellum; VL: ventral lamina; VR: ventral root; vt: vestibulum.

## Authors' contributions

SAB collected the sediment samples from Boundary Bay; generated the LM, SEM, and SSU rDNA sequence data; and wrote the first draft of the paper. NY generated the TEM data and helped with the phylogenetic analyses and interpretation of the TEM data. MH carried out the sampling, identification and LM work of the German material and helped with the identification of the Canadian material. BSL funded and supervised the collection and interpretation of the ultrastructural and molecular phylogenetic data and contributed to writing the paper. All authors have read, edited and approved the final manuscript.

## References

[B1] Leander BS Farmer MA Comparative Morphology of the Euglenid Pellicle. I. Patterns of strips and pores J Eukaryot Microbiol 2000 47 469 479 10.1111/j.1550-7408.2000.tb00076.x 11001144

[B2] Triemer RE Farmer MA Patterson DJ, Larsen J The ultrastructural organization of the heterotrophic euglenids and its evolutionary implications The Biology of Free-living Heterotrophic Flagellates 1991 Clarendon Press, Oxford 205 217

[B3] Leander BS Triemer RE Farmer MA Character evolution in heterotrophic euglenids Eur J Protistol 2001 37 337 356 10.1078/0932-4739-00842

[B4] Simpson AGB Lukes J Roger AJ The evolutionary history of kinetoplastids and their kinetoplasts Mol Biol Evol 2002 19 2071 2083 12446799 10.1093/oxfordjournals.molbev.a004032

[B5] Kivic PA Walne PL An evaluation of the possible phylogenetic relationship between euglenophyta and kinetoplastida Orig Life 1984 13 269 288 10.1007/BF00927177

[B6] Simpson AGB The identity and composition of the Euglenozoa Arch Protistenkd 1997 48 318 328

[B7] Willey RL Walne PL Kivic PA Phagotrophy and the origins of the euglenoid flagellates CRC Crit Rev Plant Sci 1988 7 303 340 10.1080/07352688809382268

[B8] Marande W Lukes J Burger G Unique mitochondrial genome structure in diplonemids, the sister group of kinetoplastids Eukaryot Cell 2005 4 1137 1146 10.1128/EC.4.6.1137-1146.2005 15947205 PMC1151984

[B9] Behnke A Bunge J Barger K Breiner HW Alla V Stoeck T Microeukaryote community patterns along an O2/H2S gradient in a supersulfidic anoxic fjord (Framvaren, Norway) Appl Environ Microbiol 2006 72 5 3626 3636 10.1128/AEM.72.5.3626-3636.2006 16672511 PMC1472314

[B10] Stoeck T Taylor GT Epstein SS Novel eukaryotes from the permanently anoxic Cariaco Basin (Caribbean Sea) Appl Environ Microbiol 2003 69 9 5656 5663 10.1128/AEM.69.9.5656-5663.2003 PMC194984 12957957

[B11] Zuendorf A Bunge J Behnke A Barger KJ Stoeck T Diversity estimates of microeukaryotes below the chemocline of the anoxic Mariager Fjord, Denmark FEMS Microbiol Ecol 2006 58 3 476 491 10.1111/j.1574-6941.2006.00171.x 17117990

[B12] Takishita K Tsuchiya M Kawato M Oguri K Kitazato H Maruyama T Genetic Diversity of Microbial Eukaryotes in Anoxic Sediment of the Saline Meromictic Lake Namako-ike (Japan): On the Detection of Anaerobic or Anoxic-tolerant Lineages of Eukaryotes Protist 2007 158 1 51 64 10.1016/j.protis.2006.07.003 16952482

[B13] Edgcomb VP Kysela DT Teske A de Vera Gomez A Sogin ML Benthic eukaryotic diversity in the Guaymas Basin hydrothermal vent environment Proc Natl Acad Sci USA 2002 99 11 7658 7662 10.1073/pnas.062186399 12032339 PMC124314

[B14] Dawson SC Pace NR Novel kingdom-level eukaryotic diversity in anoxic environments Proc Natl Acad Sci USA 2002 99 12 8324 8329 10.1073/pnas.062169599 12060775 PMC123066

[B15] Bernhard JM Buck KR Farmer MA Bowser SS The Santa Barbara Basin is a symbiosis oasis Nature 2000 403 77 80 10.1038/47476 10638755

[B16] Simpson AGB Hoff J van den Bernard C Burton HR Patterson DJ The Ultrastructure and Systematic Position of the Euglenozoon *Postgaardi mariagerensis*, Fenchel et al Arch Protistenkd 1996 147 213 335

[B17] Fenchel T Bernard C Esteban G Finlay BJ Hansen PJ Iversen N Microbial Diversity and Activity in a Danish Fjord with Anoxic Deep Water Ophelia 1995 43 45 100

[B18] Buck KR Barry JP Simpson AGB Monterey Bay Cold Seep Biota: Euglenozoa with Chemoautotrophic Bacterial Epibionts Eur J Protistol 2000 36 117 126

[B19] Yubuki N Edgcomb VP Bernhard JM Leander BS Ultrastructure and molecular phylogeny of *Calkinsia aureus*: Cellular identity of a novel clade of deep-sea euglenozoans with epibiotic bacteria BMC Microbiol 2009 9 16 10.1186/1471-2180-9-16 19173734 PMC2656514

[B20] Leander BS Keeling PJ Symbiotic Innovation in the Oxymonad *Streblomastix strix* J Eukaryot Microbiol 2004 51 291 300 10.1111/j.1550-7408.2004.tb00569.x 15218697

[B21] Leander BS Farmer MA Comparative Morphology of the Euglenid Pellicle. II. Diversity of Strip Substructure J Eukaryot Microbiol 2001 48 202 217 10.1111/j.1550-7408.2001.tb00304.x 12095109

[B22] Suzaki T Williamson RE Ultrastructure and sliding of pellicular structures during euglenoid movement in *Astasia longa *Pringsheim (Sarcomastigophora, Euglenida) J Protozool 1986 33 179 184

[B23] Leander BS Witek RP Farmer MA Trends in the evolution of the euglenid pellicle Evolution 2001 55 2215 2235 10.1111/j.0014-3820.2001.tb00737.x 11794782

[B24] Leander BS Did trypanosomatids parasites have photosynthetic ancestors? Trends Microbiol 2004 12 251 258 10.1016/j.tim.2004.04.001 15165602

[B25] Triemer RE Farmer MA An ultrastructural comparison of the mitotic apparatus, feeding apparatus, flagellar apparatus and cytoskeleton in euglenoids and kinetoplastids Protoplasma 1991 164 91 104 10.1007/BF01320817

[B26] Triemer RE Farmer MA Patterson DJ, Larsen J The ultrastructural organization of the heterotrophic euglenids and its evolutionary implications The Biology of Free-living Heterotrophic Flagellates 1991 Clarendon Press, Oxford 205 217

[B27] Roth LE An Electron-Microscope Study of the Cytology of the Protozoan *Peranema trichophorum* J Protozool 1959 6 107 116

[B28] Nisbet B An Ultrastructural Study of the Feeding Apparatus of *Peranema trichophorum* J Protozool 1974 21 39 48

[B29] Triemer RE Fritz L Structure and Operation of the Feeding Apparatus in a Colorless Euglenoid, *Entosiphon sulcatum* J Protozool 1987 34 39 47

[B30] Linton EW Triemer RE Reconstruction of the feeding apparatus in *Ploeotia costata *(Euglenophyta) and its relationship to other euglenoid feeding apparatuses J Phycol 1999 35 313 324 10.1046/j.1529-8817.1999.3520313.x 11249197

[B31] Schuster FL Goldstein S Herchenoz B Ultrastructure of a Flagellate, *Isonema nigricans *nov. gen. nov. sp., From a Polluted Marine Habitat Protistologica 1968 IV 141 149 + 5 Plates

[B32] Schnepf E Light and Electron Microscopical Observations in *Rynchopus coscinodiscivorus *spec. nov., a Colorless, Phagotrophic Euglenozoon with Concealed Flagella Arch Protistenkd 1994 144 63 74

[B33] Roy J Faktorová D Benada O Lukeš J Burger G Description of *Rynchopus euleeides *n. sp. (Diplonemea), a Free-Living Marine Euglenozoan J Eukaryot Microbiol 2007 54 137 145 10.1111/j.1550-7408.2007.00244.x 17403154

[B34] Porter D *Isonema papillatum *sp. n., a New Colorless Marine Flagellate: A Light- and Electronmicroscopic Study J Protozool 1973 20 351 356

[B35] Triemer RE Ott DW Ultrastructure of *Diplonema ambulator *Larsen & Patterson (Euglenozoa) and its Relationship to *Isonema* Eur J Protistol 1990 25 316 320 10.1016/S0932-4739(11)80123-9 23196044

[B36] Montegut-Felkner AE Triemer RE Phylogeny of *Diplonema ambulator *(Larsen and Patterson). 2. Homologies of the Feeding Apparatus Europ J Protistol 1996 32 64 76

[B37] Leander BS Esson HJ Breglia SA Macroevolution of complex cytoskeletal systems in euglenids BioEssays 2007 29 987 1000 10.1002/bies.20645 17876783

[B38] Lackey JB *Calkinsia aureus *gen. et sp. nov., a new marine euglenid Trans Am Microsc Soc 1960 79 1 105 107 10.2307/3223980

[B39] Buck KR Bernhard JM Seckbach J Protistan-Prokaryotic Symbioses in Deep-Sea Sulfidic Sediments Symbiosis: Mechanisms and Model Systems. Cellular Origin and Life in Extreme Habitats (COLE) Series 2002 4 Springer Netherlands 509 517

[B40] Leander BS Farmer MA Epibiotic bacteria and a novel pattern of strip reduction on the pellicle of *Euglena helicoideus *(Bernard) Lemmermann Europ J Protistol 2000 36 405 413

[B41] Wołowski K *Dylakosoma pelophilum *Skuja, a rare colourless euglenophyte found in Poland Algol Studies 1995 76 75 78

[B42] Fenchel T Ramsing NB Identification of sulphate-reducing ectosymbiotic bacteria from anaerobic ciliates using 16S rRNA binding ologonucleotide probes Arch Microbiol 1992 158 394 397 10.1007/BF00276298 1482269

[B43] Rosati G Seckbach J Ectosymbiosis in Ciliated Protozoa Symbiosis: Mechanisms and Model Systems. Cellular Origin and Life in Extreme Habitats (COLE) Series 2002 4 Springer Netherlands 477 488

[B44] Verni F Rosati G Peculiar Epibionts in *Euplotidium itoi *(Ciliata, Hypotrichida) J Protozool 1990 37 337 343

[B45] Rosati G Petroni G Quochi S Modeo L Verni F Epixenosomes: Peculiar Epibionts of the Hypotrich Ciliate *Euplotidium itoi *Defend Their Host against Predators J Eukaryot Microbiol 1999 46 278 282 10.1111/j.1550-7408.1999.tb05125.x

[B46] Petroni G Spring S Schleifer KH Verni F Rosati G Defensive extrusive ectosymbionts of *Euplotidium *(Ciliophora) that contain microtubule-like structures are bacteria related to Verrucomicrobia Proc Natl Acad Sci U S 2000 97 1813 1817 10.1073/pnas.030438197 PMC26518 10660683

[B47] Hoppenrath M Taxonomical and ecological investigations of flagellates from marine sands PhD thesis 2000 University of Hamburg (in German).

[B48] Uhlig G Eine einfach Methode zur Extraktion der vagilen, mesopsammalen Mikrofauna Helgol Wiss Meeresunters 1964 11 178 185 10.1007/BF01612370

[B49] Deane JA Hill DRA Brett SJ McFadden GI *Hanusia phi *gen. et sp. nov. (Cryptophyceae): characterization of '*Cryptomonas *sp. φ' Eur J Phycol 1998 33 149 154

[B50] Keeling PJ Molecular phylogenetic position of *Trichomitopsis termopsidis *(Parabasalia) and evidence for the Trichomitopsiinae Eur J Phycol 2002 38 279 286

[B51] Guindon S Gascuel O A simple, fast, and accurate algorithm to estimate large phylogenies by maximum likelihood Syst Biol 2003 52 696 704 10.1080/10635150390235520 14530136

[B52] Huelsenbeck JP Ronquist F MrBayes: Bayesian inference of phylogenetic trees Bioinformatics 2001 17 754 755 10.1093/bioinformatics/17.8.754 11524383

[B53] Cavalier-Smith T Eukaryote kingdoms: seven or nine? Biosystems 1981 14 3-4 461 81 10.1016/0303-2647(81)90050-2 7337818

